# Proof of principle study: diagnostic accuracy of a novel algorithm for the estimation of sleep stages and disease severity in patients with sleep-disordered breathing based on actigraphy and respiratory inductance plethysmography

**DOI:** 10.1007/s11325-021-02316-0

**Published:** 2021-02-16

**Authors:** Sarah Dietz-Terjung, Amelie Ricarda Martin, Eysteinn Finnsson, Jón Skínir Ágústsson, Snorri Helgason, Halla Helgadóttir, Matthias Welsner, Christian Taube, Gerhard Weinreich, Christoph Schöbel

**Affiliations:** 1 Faculty of Sleep Medicine and Telemedicine, University Medicine Essen - Ruhrlandklinik, West German Lung Center, University Duisburg-Essen, Duisburg, Germany; 2 Department of Pulmonology, University Medicine Essen - Ruhrlandklinik, West German Lung Center, University Duisburg-Essen, Duisburg, Germany; 3 Nox Research, Nox Medical ehf, Reykjavík, Iceland

**Keywords:** Sleep stage estimation, Artificial intelligence, Recurrent neural network, Actigraphy, RIP

## Abstract

**Purpose:**

In this proof of principle study, we evaluated the diagnostic accuracy of the novel Nox BodySleep^TM^ 1.0 algorithm (Nox Medical, Iceland) for the estimation of disease severity and sleep stages based on features extracted from actigraphy and respiratory inductance plethysmography (RIP) belts. Validation was performed against in-lab polysomnography (PSG) in patients with sleep-disordered breathing (SDB).

**Methods:**

Patients received PSG according to AASM. Sleep stages were manually scored using the AASM criteria and the recording was evaluated by the novel algorithm. The results were analyzed by descriptive statistics methods (IBM SPSS Statistics 25.0).

**Results:**

We found a strong Pearson correlation (*r*=0.91) with a bias of 0.2/h for AHI estimation as well as a good correlation (*r*=0.81) and an overestimation of 14 min for total sleep time (TST). Sleep efficiency (SE) was also valued with a good Pearson correlation (*r*=0.73) and an overestimation of 2.1%. Wake epochs were estimated with a sensitivity of 0.65 and a specificity of 0.59 while REM and non-REM (NREM) phases were evaluated a sensitivity of 0.72 and 0.74, respectively. Specificity was 0.74 for NREM and 0.68 for REM. Additionally, a Cohen’s kappa of 0.62 was found for this 3-class classification problem.

**Conclusion:**

The algorithm shows a moderate diagnostic accuracy for the estimation of sleep. In addition, the algorithm determines the AHI with good agreement with the manual scoring and it shows good diagnostic accuracy in estimating wake-sleep transition. The presented algorithm seems to be an appropriate tool to increase the diagnostic accuracy of portable monitoring. The validated diagnostic algorithm promises a more appropriate and cost-effective method if integrated in out-of-center (OOC) testing of patients with suspicion for SDB.

**Supplementary Information:**

The online version contains supplementary material available at 10.1007/s11325-021-02316-0.

## Introduction

Sleep-disordered breathing (SDB) shows a high prevalence in the general population [1]. SDB leads to repetitive arousals and activation of the sympathetic nervous system resulting in surges of blood pressure and heart rate and stressing the cardiovascular system. The long-term risk of developing cardiovascular diseases is significantly increased [1]. Affected patients often complain of snoring, non-restorative sleep, or daytime sleepiness. This can increase the risk of road traffic accidents [2]. Current epidemiological studies show a high prevalence of SDB. In Germany, for instance, 29.7% of all men and 13.2% of all women are suspected to suffer from moderate to severe sleep apnea. If adding those with low-grade sleep apnea, the prevalence figures are 59.4% and 33.2% respectively [3]. However, therapy for mild sleep apnea is only necessary in the case of cardiovascular risk or associated daytime sleepiness [4]. Due to limited diagnostic capacities, a high number of unrecognized cases have to be assumed. An undetected and therefore untreated SDB is associated with high direct and indirect costs [5]. The economic implications of SDB are strongly underestimated [6]. In 2016, Frost and Sullivan showed that, for example in the USA, the estimated burden of cost for undiagnosed OSA was $149.6 billion. They estimated that an additional $49.5 billion would be necessary to diagnose and treat every American adult who has OSA [7]. In clinical routine, a patient with suspicion for SDB will get a portable monitoring for a one-night examination of respiratory parameters in the home environment. In general, most portable monitoring systems are not able to assess sleep time; numbers of apneas or hypopneas are related to recording time leading to an overall underestimation of real SDB-severity. Thus, in the case of inclusive findings in portable monitoring (e.g., not relevant increased AHI), guidelines recommend a full polysomnography (PSG) in a sleep lab as diagnostic gold standard. Currently, many patients are sent for an in-center PSG who could otherwise have been diagnosed in a more accurate way by a more sophisticated home sleep testing. Smart diagnostic devices integrating automatic data processing algorithms could enable a more accurate detection of SDB in a home setting without a time, personnel, and cost consuming PSG examination. Undoubtedly, PSG is a necessary tool for patients with complex comorbidities for an accurate clinical diagnosis. Thus, using new diagnostic approaches could lead to a more efficient exploitation of existing sleep lab capacities for patients really requiring those resources. An increase of diagnostic accuracy of home sleep testing by smarter diagnostic techniques compared to gold standard PSG could help to narrow the gap between the high prevalence of SDB and limited diagnostic capacities.

In this proof of principle study, we analyzed an artificial intelligence (AI)–based algorithm to differentiate between wake, rapid eye movement sleep (REM), and non-rapid eye movement (NREM) sleep based on features extracted from actigraphy and respiratory inductance plethysmography (RIP) belts (Nox Medical, Iceland). The algorithm presented here is a machine learning (ML) algorithm based on an artificial recurrent neural network (RNN) trained using supervised learning. The RNN contains three fully connected dense layers that feed into a recurrent layer. This method may significantly simplify the diagnostic pathway for SDB by allowing the clinician to differentiate between sleep states without collecting and analyzing data with full PSG equipment. Additionally, the use of this algorithm may help to increase the diagnostic accuracy for the estimation of sleep time resulting in a more accurate estimation of sleep-related parameters like apnea-hypopnea index (AHI), total sleep time (TST), or sleep efficiency (SE).

## Methods

### Patients

Between October 2019 and January 2020, we included 128 patients referred to our sleep lab due to suspected sleep-disordered breathing. We excluded all pregnant women according to the declaration of Helsinki (2013). We excluded 17 patients due to artifacts in one or more PSG channels.

This study was approved by the ethics committee of the University Duisburg-Essen and all participating subjects provided informed consent (19-8963-BO).

### Polysomnography

All study patients were examined by means of a digital polysomnography (Nox A1®, Nox Medical, Iceland) including electroencephalography (EEG), electrooculography (EOG), electromyography (EMG) of submental and tibialis muscles, rib cage and abdominal Respiratory Inductance Plethysmography (RIP), pulse oximetry (Nonin 3150, Minnesota, USA), measurement of respiratory flow by nasal cannula at a sample frequency of 200 Hz and body position [8, 9].

Apnea and hypopnea events were scored according to AASM 2012 [10].

### Nox BodySleep^TM^ 1.0 algorithm

**Fig. 1:**
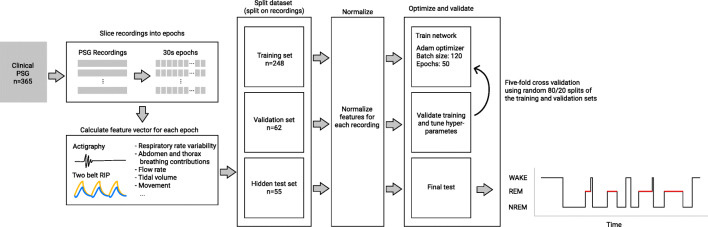
The BodySleep^TM^ 1.0 network architecture. Connections between nodes are not drawn; the network is fully connected during prediction. Twenty-two percent dropout was used during training for regularization. The time step (*n*) refers to the epoch number of the study being analyzed

The Nox BodySleep^TM^ 1.0 (Nox Medical, Island) algorithm is a machine learning algorithm utilizing a feature-based artificial neural network to classify 30 s epochs into sleep states. The neural network is comprised of three dense layers, each with 70 nodes, followed by a recurrent layer with 50 gated recurrent units (GRU) blocks. The classification layer consists of 3 nodes, representing each of the three sleep states: wake, REM, and NREM. A SoftMax function is used to select the class by selecting the output of the classification layer with the highest score. The activation function used for the hidden layers and the gated recurrent unit (GRU) is the so-called rectifier or Rectified Linear Unit (ReLu) function (Fig. 1). The ReLu function is depicted showing that negative inputs result in an output value of zero, while positive inputs result in an output value equal to the input. The GRU is a special mechanism used in artificial neural networks to introduce an element which can retain a memory of previous inputs to be used with current inputs for prediction. The GRUs act as a sort of memory elements in the artificial neural networks.

The Nox BodySleep^TM^ 1.0 algorithm training, validation, and testing data consisted of 365 manually scored sleep studies originating from two sources. The 365 studies were split into a training and a validation set of 310 studies as well as a hidden test set of 55 studies. The hidden test set was only used for the final internal validation of the model before it was implemented into the Noxturnal software and used for the current external validation. The 310 studies used for training and validation were randomly split into training and validation sets with an 80/20 split during 5-fold cross validations during the model development. During training, a drop out of 0.22 was used in each of the three dense and the GRU layers for regularization. The three output classes were given different weights accounting for their prevalence in the training set: wake 1, REM 0.7, and NREM 0.6.

The input signals to the algorithm are the abdominal and thoracic RIP signals, respiratory rate and activity. Features such as amplitude, standard deviation, changes in amplitude, signal correlations, breathing durations, and other statistical metrics were derived from the signals. The features were normalized in a robust way, subtracting the median and dividing by the inter quartile range for each study.

When using the algorithm, the same features are calculated and the normalization is performed for each study. The model input consists of the features calculated for 25-time steps, where each timestep represents a 30 s sleep epoch. The output is taken as the predicted sleep state of the 23^rd^ time step. Therefore, of the 25 input timesteps, 22 represent past epochs, 1 represents the current epoch of interest, and 2 represent future epochs.

**Fig. 2:**
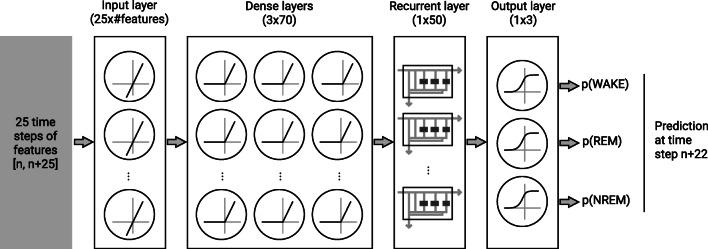
The training and validation pipeline for the BodySleep^TM^ 1.0 algorithm

During training, the Adam optimizer was used with categorical cross entropy loss. The model was trained with a batch size of 120 over 50 training epochs. The learning rate was set to 0.0001 (Fig. 2).

We tested the diacnostic accuracy of the novel Nox BodySleep^TM^ 1.0 algorithm for the estimation of sleep based on actigraphy and RIP belts. Validation was performed against manually scored polysomnography recordings.

## Statistics

Methods of descriptive statistics (frequency, mean ± standard deviation, range, sensitivity, and specificity) were used for the analysis of sleep variables. We performed Pearson correlation analysis and constructed Bland-Altman plots. The Mann-Whitney *U* test was used to analyze the significance of the agreement of the examined distributions. Additionally, Cohen’s kappa, which measures the agreement between two raters or methods, as well as sensitivity and specificity, was calculated. For the analysis of differences between different subgroups, the Kruskal-Wallis test was conducted. Statistical analysis was performed using SPSS 22.0 (IBM SPSS Statistics, Armonk, New York, USA). A *p* value ≤ 0.05 was considered to be statistically significant.

## Results

Our study population consisted of 128 patients in total (79 men, 49 women, median age 62±13 years). Table 1 depicts patient characteristics. All included patients showed sleep-disordered breathing. The presence of another sleep disorder was excluded by the use of questionnaires as well as the examination by an experienced sleep physician. Furthermore, there was no hint for another sleep disorder according to polysomnographic results. Both diagnostic and therapeutic nights were included in the assessment.

**Table 1:** Patient characteristics (*n*=128) of the studied cohort (BMI: body mass index; AHI: apnea-hypopnea index; ODI: oxygen desaturation index)

Metrics	Mean	Std	Min	Max
Age [years]	61.5	13.4	18	86
Height [cm]	173.8	9.9	149	196
Weight [kg]	99.2	24.0	49	180.0
BMI [mg/m^2^]	31.0	7.2	18	58.8
AHI [/h]	19.0	18.8	0	84.3
ODI [/h]	21.3	19.6	0	92.1
Analysis duration [min]	304.4	261.1	288	510.25

**Fig. 3:**
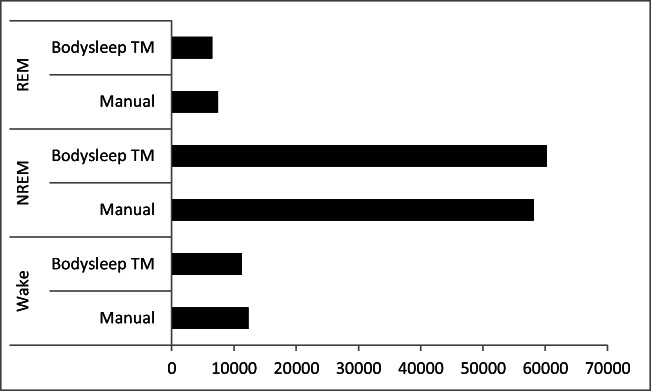
Comparison of the number of scored epochs via Nox BodySleep^TM^ 1.0 versus manual scoring of 128 sleep lab patients

We analyzed a total of 77,940 epochs of 30 s in length. The comparison between manual scored epochs and epochs scored by Nox BodySleep^TM^ 1.0 is shown in Fig. 3. We obtained a Cohen’s kappa of 0.62, indicating a substantial agreement between the Nox BodySleep^TM^ 1.0 and the manual scoring.

Additionally, we analyzed the diagnostic accuracy, represented by sensitivity and specificity, of the Nox BodySleep^TM^ 1.0 sleep/wake estimation compared to an experienced technician who manually scored sleep/wake stages for a total of 128 patients (Table 2).

**Table 2:** Sensitivity and specificity of sleep stage estimation by Nox BodySleep^TM^ 1.0

Stage	Sensitivity	Specificity
Wake	0.65	0.59
REM	0.72	0.68
NREM	0.74	0.70
Average	0.70	0.66

We also calculated Pearson correlation coefficient *r* and the bias for estimation of AHI, SE, and TST by Nox BodySleep^TM^ 1.0 for four different AHI-groups. Group 1 includes patients with an AHI below 5 events/h, group 2 includes the range of 5–15 events/h, while group 3 includes the AHI between 15 and 30 events/h and group 4 includes all patients with an AHI above 30/h.

**Fig. 4:**
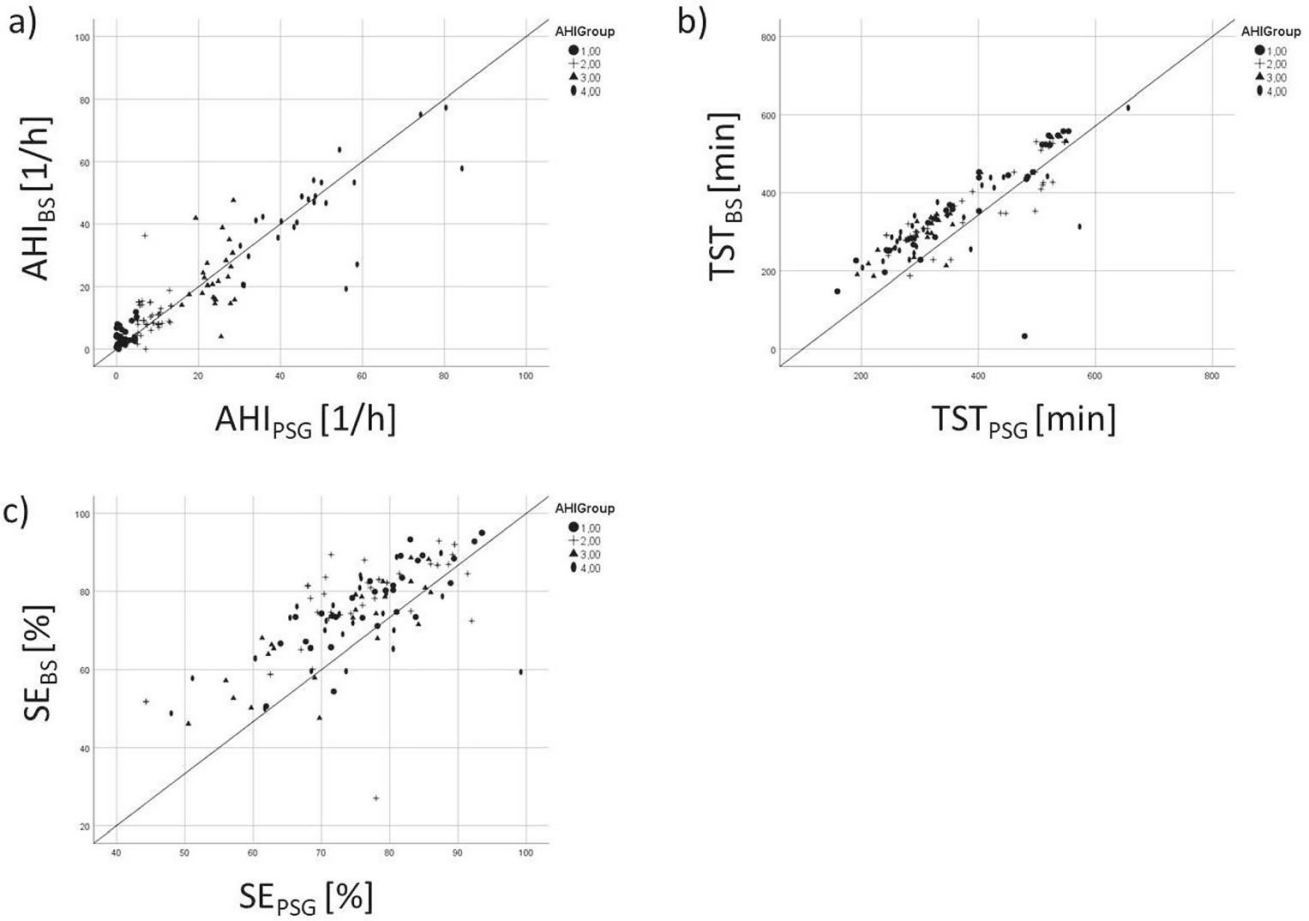
Scatter plot of **a** AHI, **b** TST, and **b** SE

Results are summarized in Fig. 4 and Table 3.

**Table 3:** Pearson correlation for the analyzed AHI subgroups. *p<0.005, **p<0.001. AHI, apnoea hypopnoea index; TST, total sleep time; SE, sleep efficiency

	AHI group 1	AHI group 2	AHI group 3	AHI group 4	Overall	Kruska-Wallis *p*
TST [min]	0.79**	0.87**	0.85**	0.63**	0.81*	0.26
SE [%]	0.84**	0.60**	0.84**	0.588**	0.73*	0.01

Comparing AHI scored by BodySleep™1.0 algorithm with PSG-based manually scored AHI, we found an overall correlation of *r*=0.91 with an overestimation of 0.2/h, received by Bland-Altman analysis (Supplementary Material). Additionally, we found an overall correlation of *r*=0.73 with an overestimation of 2.1% for sleep efficiency (SE) and a correlation of *r*=0.81 with an overestimation of 14 min for total sleep time (TST), respectively.

To analyze diagnostic accuracy of BodySleep™1.0 algorithm depending on different severity grades of SDB, we divided the sample into 4 subgroups based on manually scored AHI values: AHI group 1 with AHI <5/h showing no relevant SDB, AHI group 2 with AHI between 5/h and 15/h representing mild graded SDB, AHI group 3 with AHI between 15 and 30/h showing moderate graded SDB and AHI group 4 with AHI≥30/h representing severe graded SDB.

Total sleep time is estimated to be of good diagnostic quality in all investigated subgroups, which is also confirmed by the Kruska-Wallis analysis.

Sleep efficiency is more likely to be overestimated by the BodySleep^TM^ 1.0 in patients with mild and severe sleep apnea than in healthy subjects or patients with moderate sleep apnea. This difference is shown to be significant in the Kruska-Wallis analysis (Table 3).

The cross-sectional analysis of the AHI subgroups resulted in the BodySleep^TM^1.0 classifying patients into the analyzed AHI threshold groups with strong sensitivities and specificities (Table 4).

**Table 4:** Sensitivity and specificity for the analyzed AHI subgroups

AHI group	Sensitivity [%]	Specificity [%]	*N*
1	72.7	91.0	33
2	85.3	92.8	41
3	72.7	97.2	26
4	72.4	92.1	28

We also examined the diagnostic accuracy of the BodySleep^TM^ 1.0 is for estimating the sleep states in order to identify wake-sleep transitions. For this purpose, we searched for the first epoch scored as sleep in the manually analyzed PSGs. From this epoch, we went ten epochs forward and backward and compared these epochs with the BodySleep^TM^ 1.0 scoring results using four fields chart analysis. We found a sensitivity of 81%, a specificity of 96%, and a F1 score of 74%, indicating that the BodySleep^TM^ 1.0 estimates the first occurrence of sleep during waking-sleep transition with good clinical accuracy.

## Discussion

Due to the high prevalence of SDB and related diseases as well as limited diagnostic capacities, the development and validation of novel technologies like BodySleep™ 1.0 will enable a more accurate out of center (OOC) testing and is a major research priority in sleep medicine.

This is the first study to evaluate the diagnostic accuracy of Nox BodySleep^TM^ 1.0, a novel AI-based algorithm for the estimation of sleep states, for the determination of AHI and of sleep quality parameters (TST and SE) in different AHI subgroups.

First, we found a substantial agreement between Nox BodySleep^TM^ 1.0 scored epochs with manually scored epochs and a strong Cohen’s Kappa of 0.62.

Second, we observed an excellent agreement for AHI estimation (*r*=0.91) and an appropriate agreement between manually scored PSG and Nox BodySleep^TM^ 1.0^TM^ for the estimation of TST and SE with *r*=0.81 and *r*=0.73, respectively.

The BodySleep^TM^ 1.0 seems to be a convenient algorithm for OOC testing as it detects different, clinically relevant AHI thresholds with high diagnostic accuracy. In addition, the BodySleep^TM^ 1.0 also estimates first occurrence of sleep with good diagnostic accuracy leading to an improved diagnostic value of outpatient portable monitoring.

However, our findings should be discussed with existing evidence on diagnostic approaches using AI mechanisms. Regarding automatic sleep/wake stage classification in patients with obstructive sleep apnea, Ucar et al. [11] analyzed the diagnostic accuracy of an algorithm for automatic sleep staging based on heart rate variability (HRV) derived from photoplethysmography (PPG) by feature extraction followed by the *k*-nearest neighbors classification and support vector machines (SVM) on a total of 10 patients. They found an accuracy of 73.36%, a sensitivity of 0.81, a specificity of 0.77, a Cohen’s kappa of 0.59, and an *F*-measurement of 0.79, indicating the strong clinical accuracy of the presented algorithm. This is comparable to the results gained with the presented 3 class classification algorithm.

Korkalainen et al. [12] presented a deep learning algorithm for sleep staging based on PPG data in 894 patients with suspected sleep-disordered breathing. They analyzed a three-stage (wake/NREM/REM), a four-stage (wake/N1+N2/N3/REM), and a five-stage (wake/N1/N2/N3/REM) model with strong epoch-by-epoch accuracies of 80.1%, 68.5%, and 64.1%, respectively, resulting in a moderate agreement compared to manual EEG scoring. Additionally, this deep learning algorithm estimated clinical parameters like total sleep time (TST), sleep efficiency (SE), sleep stage percentage, and AHI with low bias. This algorithm is superior to the one presented here, because it allows a finer distinction up to the 5 class classification. It is to be discussed whether the Bodysleep^TM^ 1.0 can achieve an even better performance by adding further channels or features, for example, by using the HRV. A study of Fonseca et al. [13] investigated a machine-learning based sleep-staging method using HRV features derived from PPG and features from the RIP belts. They found a strong accuracy of 80% for the abovementioned three-class task as well as a slight overestimation of TST and SE of 13.4 min and 2.9%, respectively. Additionally, they found a high inter-individual variability regarding the accuracy of the algorithm. This is comparable to the results of the algorithm investigated in this study.

Beattie et al. [14] also presented an automated algorithm for sleep stage estimation based on PPG and accelerometry features in a total of 60 adults. They found a moderate overall accuracy of 59% and a Cohen’s kappa of 0.52 and no observable bias for the estimation of the different sleep stages’ durations. In comparison, the Bodysleep^TM^ 1.0 shows a better clinical accuracy. A study of Motin et al. [15] investigated a PPG-based supervised Machine learning sleep-wake classification algorithm using SVMs with cubic kernel. They extracted time-domain features from PPG signals and PPG-based surrogate values for cardiac parameters to classify wake and sleep stages, resulting in a strong accuracy of 81.10%, a sensitivity of 81.06%, a specificity of 82.50%, a precision of 99.37%, and an *F* score of 81.74%, indicating high diagnostic quality. This is also consistent with our results, showing that the Bodysleep^TM^ 1.0 is comparable to the other 3 class classification algorithms in terms of performance and clinical accuracy.

Another study of Fonseca et al. [16] dealed with an automatic sleep staging based on heart rate variability and body movements. They found a substantial agreement of the algorithm with four-class sleep staging with a Cohen’s kappa of 0.60 and an accuracy of 75.9%. For sleep/wake classification, they found a Cohen’s kappa of 0.65 and a sensitivity to wake of 72.8% and a sensitivity of 94.0%, which is consistent to the results presented here. BodySleep^TM^ 1.0

Lyon et al. [17] used a sonar smartphone technology app to estimate sleep states and to screen for SDB patterns based on respiration and movement in a database including 94 overnight measurements. They found a sensitivity of 94% and a specificity of 97% for an AHI threshold above 15/h. These values are comparable to the AHI estimations gained by Bodysleep^TM^ 1.0.

Schade et al. [18] presented a consumer-marketed non-contact device which algorithm estimates sleep states (5-class classification) based on movements and respiration. They found an overall accuracy of 87%, a sensitivity for sleep state of 96%, and a sensitivity for wake of 73% as well as good estimations for sleep-related parameter like TST or wake after sleep onset (WASO). The performance of this algorithm is slightly better than the one of the algorithm presented here.

Yang et al. [19, 20] presented a sleep state estimation algorithm for wake, rapid eye movement (REM), and non-REM (NREM) sleep detection (3-class classification) using two respiratory variability (RV) features extracted from oro-nasal airflow signals provided in the sleep-EDF database. They found an overall accuracy of 74.0% and Cohen’s kappa coefficient of 0.49, indicating that this algorithm has a comparable diagnostic accuracy to the Bodysleep^TM^ 1.0 algorithm presented here.

A current study of Lauteslager et al. [10] aims to evaluate the sleep staging performance of the radar-based Circadia Contactless Breathing Monitor (model C100) and its underlying sleep analysis algorithm on a group of healthy sleepers. They trained the algorithm on PSG data obtained in the initial dataset (*n*=17), and validated it using leave-one-subject-out cross-validation. An epoch-by-epoch accuracy of 75.0%, 59.9%, 74.8%, and 57.1% was found for “deep,” “light,” “REM,” and “wake” respectively, indicating that this algorithm solves the 4-class classification problem with good clinical accuracy. Compared to this algorithm, Bodysleep^TM^ 1.0 performed slightly weaker, as it currently leaks the distinction between light and deep NREM sleep.

Table 5 summarizes the results of abovementioned reference studies compared with our study results regarding different classes of sleep-stage-discrimination.

**Table 5:** AI-based study results regarding different classes of sleep-stage-discrimination

	Class	Accuracy [%]	Sensitivity [%]	Specificity [%]	Cohen’s kappa
Ucar et al. [11]	3	73.4	81.0	77.0	0.59
Kokalinen et al. [12]	5	80.1			
Fonseca et al. [13]	3	80.0			
Beattie et al. [14]	3	59.0			0.52
Montin et al. [15]	3	81.1	81.1	82.5	
Schade et al. [18]	5	87.0	96.0		
Yang et al. [19, 20]	3	74.0			0.49
BodySleep^TM^ 1.0	3	73.0	75.7	93.3	0.62

The presented method has an important impact as the evaluation of sleep does not require EEG and EMG, since it is solely based on the evaluation of physiological parameters affected by sleep. A portable monitoring with such integrated algorithm would be much more comfortable for the patient compared to the full PSG setting. Furthermore, it could be less error-prone, as no electrodes can slip or get lost. While showing a comparable diagnostic accuracy to the algorithms presented above, BodySleep^TM^ 1.0 algorithm does not include data from PPG. Therefore, this algorithm is independent of signal quality of pulse oximetry which is often disturbed by loss of contact during outpatient recordings.

However, we have to acknowledge some limitations. Nox BodySleep^TM^ 1.0 lacks heart rate variability analysis which could reflect autonomic changes during disturbed sleep. Therefore, we propose the inclusion of heart rate variability in the neural network of the Nox BodySleep^TM^ 1.0 algorithm, as this is likely to significantly increase the diagnostic accuracy. Additionally, the current algorithms should be adapted in order to get more appropriate diagnostic accuracy also for patients with other sleep disorders like periodic limb movement disorder (PLMD).

In summary, Nox BodySleep^TM^ 1.0 algorithm can the diagnostic value of outpatient portable monitoring by estimating sleep in an appropriate way leading to a more reliable finding of apnea-hypopnea index. The diagnostic accuracy is comparable to other validated algorithms. Nox BodySleep^TM^ 1.0 is easy to use and could help to increase the diagnostic accuracy of home sleep testing promising a more efficient and economic diagnostic pathway.

## Conclusion

The algorithm shows a moderate diagnostic accuracy for the estimation of sleep. In addition, the algorithm determines the AHI with a good agreement to the manual scoring and it shows a good diagnostic accuracy in estimating wake-sleep transition. The presented algorithm seems to be an appropriate tool to increase the diagnostic accuracy of portable monitoring. The validated diagnostic algorithm promises a more appropriate and cost-effective method if integrated in Out-of-center (OOC) testing of patients with suspicion for SDB.

## Supplementary Information

ESM 1 (DOCX 146 kb)

## Abbreviations

AASM: American Academy of Sleep Medicine
AHI: Apnoea hypopnoea index
AI: Artificial intelligence
BMI: Body mass index
EEG: Electroencephalogram
EMG: Electromyography
EOG: Electrooculography
ICC: Interclass correlation coefficient
ML: Machine learning
NREM: Non REM
N1: NREM 1 sleep stage
N2: NREM 2 sleep stage
N3: NREM 3 sleep stage
OOC: Out-of-center
OSA: Obstructive Sleep Apnea
PG: Polygraphy
PPG: Photoplethysmography
PSG: Polysomnography
REM: Rapid eye movement sleep
RIP: Respiratory inductance plethysmography
RNN: Recurrent neural network
SDB: Sleep-disordered breathing
SVM: Support vector machines
US: United States
W: Wake
