# Supplementary material for: Proof of principle study: diagnostic accuracy of a novel algorithm for the estimation of sleep stages and disease severity in patients with sleep-disordered breathing based on actigraphy and respiratory inductance plethysmography
Source: Sleep Breath. 2021 Feb 16;25(4):1945–52. doi: 10.1007/s11325-021-02316-0 (PMC8590674; doi:10.1007/s11325-021-02316-0)
Supplement: Supplementary file 1 — (DOCX 146 kb) [file 11325_2021_2316_MOESM1_ESM.docx]

Supplemental Material

1. Bland-Altman Analysis


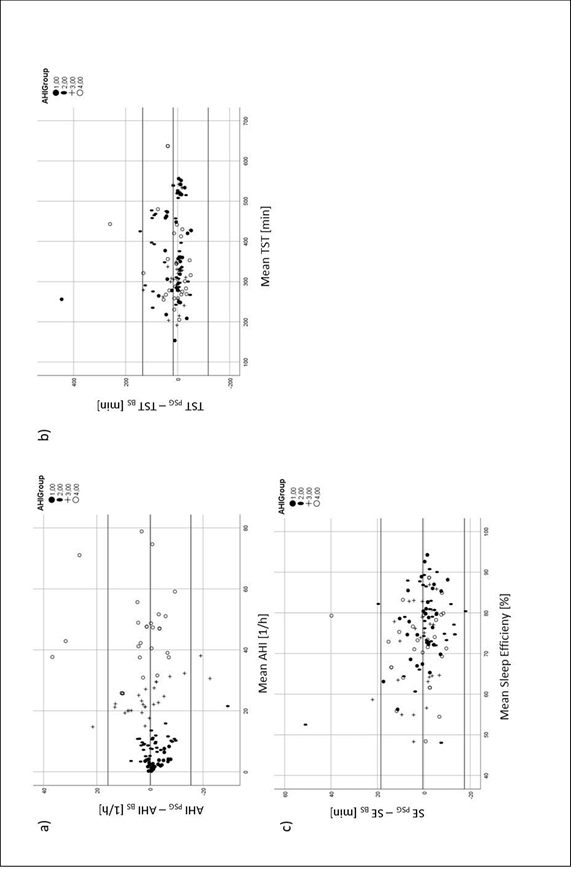


*Figure 6. Bland-Altman-analysis of a) AHI b) TST and b) SE.*

Additionally we performed a Bland-Altman analysis (figure 6), which confirmed the results of the correlation analysis. The outliers seen in the Bland-Altman analysis are patients who had asymptomatic, comorbid periodic leg movement disorder firstly diagnosed in polysomnography.

1. ROC Analysis

| Stage | Sensitivity | Specificity | AUC | Asympt. Significance |
| --- | --- | --- | --- | --- |
| Wake | 0.65 | 0.59 | 0.623 | 0.000 |
| REM | 0.72 | 0.68 | 0.715 | 0.000 |
| NREM | 0.74 | 0.70 | 0.751 | 0.001 |
| Average | 0.7 | 0.66 |  |  |


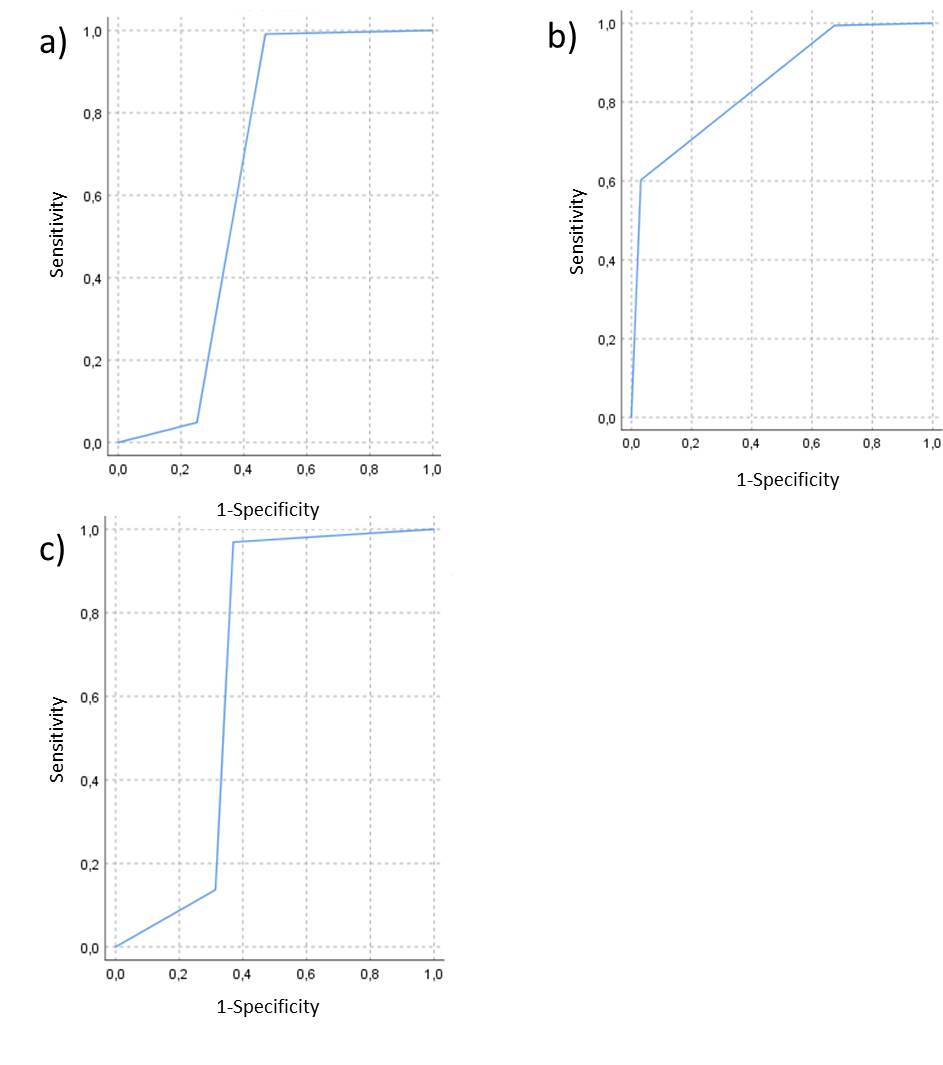


*Figure 4. ROC analysis of diagnostic accuracy of sleep stage estimation of Nox BodySleep^TM^ 1.0 versus manual scoring of PSG regarding a) wake, b)NREM and c) REM stage.*
